# L-phenylalanine in potato onion (*Allium cepa* var. *aggregatum* G. Don) root exudates mediates neighbor detection and trigger physio-morphological root responses of tomato

**DOI:** 10.3389/fpls.2023.1056629

**Published:** 2023-02-16

**Authors:** Hongjie Yu, Danmei Gao, Muhammad Khashi u Rahman, Shaocan Chen, Fengzhi Wu

**Affiliations:** ^1^ Institute of Agricultural Economy and Scientific Information, Fujian Academy of Agricultural Sciences, Fuzhou, China; ^2^ College of Horticulture and Landscape Architecture, Northeast Agricultural University, Harbin, China; ^3^ Key Laboratory of Cold Area Vegetable Biology, Northeast Agricultural University, Harbin, China

**Keywords:** interspecific interaction, root exudates, chemical cue, root gravitation, tomato

## Abstract

**Interaction:**

Despite numerous recent insights into neighbor detection and belowground plant communication mediated by root exudates, less is known about the specificity and nature of substances within root exudates and the mechanism by which they may act belowground in root-root interactions.

**Methods:**

Here, we used a coculture experiment to study the root length density (RLD) of tomato (*Solanum lycopersicum* L.) grown with potato onion (*Allium cepa* var. *aggregatum* G. Don) cultivars with growth-promoting (S-potato onion) or no growth-promoting (N-potato onion) effects.

**Results and Discussion:**

Tomato plants grown with growth-promoting potato onion or its root exudates increased root distribution and length density oppositely and grew their roots away as compared to when grown with potato onion of no growth-promoting potential, its root exudates, and control (tomato monoculture/distilled water treatment). Root exudates profiling of two potato onion cultivars by UPLC-Q-TOF/MS showed that L-phenylalanine was only found in root exudates of S-potato onion. The role of L-phenylalanine was further confirmed in a box experiment in which it altered tomato root distribution and forced the roots grow away. *In vitro* trial revealed that tomato seedlings root exposed to L-phenylalanine changed the auxin distribution, decreased the concentration of amyloplasts in columella cells of roots, and changed the root deviation angle to grow away from the addition side. These results suggest that L-phenylalanine in S-potato onion root exudates may act as an “active compound” and trigger physio-morphological changes in neighboring tomato roots.

## Introduction

Plants were traditionally considered passive entities, interacting with neighbors for belowground resources only (Pierik et al., 2013). However, an ever-increasing body of work from the last few decades completely overturned the concept and revealed that plants actively detect, respond and interact with neighboring plants and the environment, and the involved mechanisms are far more complex than they seem (Bilas et al., 2021). For instance, plant root system exhibit great plasticity in architecture in response to the external stimuli to optimize growth patterns (Malamy, 2005; Sun et al., 2007). Although, our knowledge of interplant interactions in natural systems has greatly improved recently, the same in agricultural systems remains insufficient. Intercropping, a farming practice involving two or more crop species or genotypes growing together and coexisting simultaneously (Li et al., 2014), can offer a higher land and resource utilization efficiency, efficient economic benefits, and environmental stability (Yu et al., 2017; Zhou et al., 2019; Jin et al., 2022; Zhou et al., 2022). Previous studies have suggested that intercropping improves biomass and yield of crops by changing their root architecture and distribution (Yu et al., 2017; Ding et al., 2021). However, a complete mechanism about how plants in agricultural systems mediate neighbor detection and cause the changes in adjacent plant root system remain unclear.

Plants exude a significant amount of organic compounds into the soil, which plays a wide range of functional roles such as signaling between plants to their surrounding environments (Wang et al., 2021). Plants can detect various compounds exuded by neighboring plants and can respond with alteration in phenotypic traits (Wang et al., 2021). These exudates might thus provide the key information about the proximity of neighboring plants, and their phylogenetic and physiological status, and may allow roots to precisely avoid, attract, or stay neutral in response to neighboring roots (Hodge, 2009). For example, coumarin, flavonoids, and glucose in root exudates of the neighboring plant can affect the growth, movement, and gravity sensing of plant roots (Lupini et al., 2013; Singh et al., 2014). Plant release of chemicals to change the growth, development, survival, and reproduction of neighboring plants (Bilas et al., 2021) is an important aspect of interplant interaction. Different root behavioral responses of focal plants to root secreted substances from neighbors have been reported (Semchenko et al., 2014; Kong et al., 2018). Root exudates of different plant species or cultivars can carry different recognition information, and therefore adding root exudates of the different plants around the roots of homologous plants lead to changes in specific root length, root branch density, root biomass, and root length density (RLD, referring to the total length of roots per unit volume of soil) (Biedrzycki et al., 2010; Semchenko et al., 2014). However, the specificity and nature of substances in root exudates that mediate changes in neighboring plant root architecture and the possible mechanisms remain largely unknown.

Plant root distribution is closely related to plant growth and nutrient absorption capacity, which can be influenced by a variety of environmental factors, including nutrients, water, and soil microorganisms (Malamy, 2005; Osmont et al., 2007; Contreras-Cornejo et al., 2009; Felten et al., 2009; Zamioudis et al., 2013). The existence of gravity is related to the formation of plant root distribution, and the gravity magnitude and direction can directly affect the development, stress response, and other activities of the root system (Cardoso et al., 2007). It is known that the perception of gravity is mediated by the deposition of amyloplast in the columella cells of the root tips, which provides clues and advice to the root system about the growth direction (Sato et al., 2014). Meanwhile, lateral transport of auxin is induced by gravity stimulation, which promotes the formation of auxin gradient in roots. The accumulation side of auxin inhibits cell elongation and causes root bending (Hasegawa et al., 2017). Therefore, the study on the deposition of amyloplasts and the distribution of auxin in root tip corpuscle cells is important for understanding the mechanism of plant-plant root interaction.

Tomato (*Solanum lycopersicum* L.), a vegetable usually cultivated in the greenhouse, is an important crop cultivated globally. The tomato root system developed, easy to produce side roots. Among 47 cultivars of potato onion (*Allium cepa* L. var. *agrogatum* G.Don.) screened from Jilin and Heilongjiang provinces in China, the cultivar “Suihua” (S-potato onion) has the strongest growth-promoting effect, while “Ningan Hongcheng” (N-potato onion) has been found with no obvious influence on neighbors and possess no growth-promoting effect (Liu et al., 2013; Yu et al., 2017). Previously, we have found that companion cropping with S-potato onion altered the root distribution of the tomato plant (Fu et al., 2016; Yu et al., 2017). However, the underlying mechanisms that mediate such interplant interaction remain unclear. Based on our previous findings, we hypothesize that: (1) the change in tomato root distribution is caused by some specific “active substances” in root exudates of potato onion, and therefore, the root exudates profile of two potato onion cultivars would be different; and (2) those substances affected the gravity of tomato by changing the deposition of amyloplast and the distribution of auxin in the root tip, and thus changed the distribution direction of tomato root. To test the hypotheses, first, we studied the root response of tomatoes by coculturing with two potato onion cultivars or their exogenously applied root exudates. Then, we used UPLC-MS for root exudates profiling to identify the distinct specific compounds in root exudates of both cultivars that could potentially mediate plant interspecific interaction. Finally, we verified that the effect of root exuded “active substances” on tomato root gravitropic responses are related to the amyloplast synthesis pathway and auxin transportation-related gene expression.

## Results

### The root length density of tomato cocultured with two cultivars of potato onion

**Figure 1:**
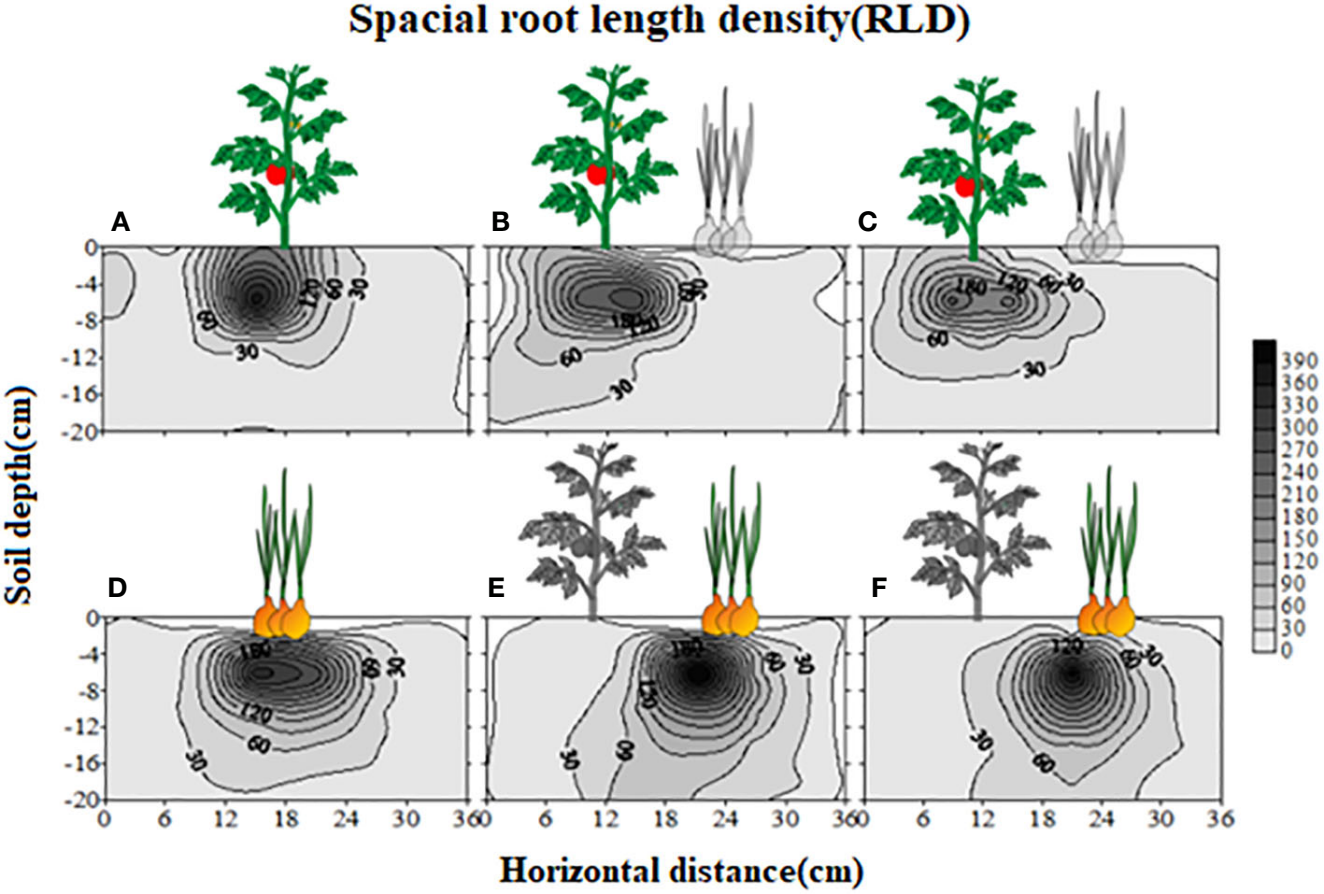
Effects of different interaction modes on the root length density (RLD) distribution of tomato and potato onion. **(A)** sole tomato; **(B)**: tomato grown with S-potato onion; **(C)** tomato grown with N-potato onion; **(D)** sole potato onion; **(E)** S-potato onion grown with tomato; **(F)** N-potato onion grown with tomato. Colored plants are those whose root distribution is shown in the corresponding panel, while plants in dark show the positions of companion cropped species. The scale represents the special root length density, and the shading density indicates the root intensity.

Coculture with S-potato onion made the tomato roots (30cm/120cm^3^ and 60cm/120cm^3^ of root length density) grow away from potato onion as compared to when grown with N-potato onion where tomato roots were not distracted and had almost even distribution (**Figure 1**). Compared with tomato monoculture (**Figure 1A**), the tomato plant grown with S-potato onion increased its root distribution range by 6 cm in the vertical direction with the root length density of 30cm/120cm^3^, and the root distribution range on no coculture side was larger than coculture side (**Figure 1B**). Although tomato cultivated with N-potato onion also increased the root vertical distribution by 3 cm (about 15cm deeper in coculture with N but about 12cm in control) with 30cm/120cm^3^ of root length density, unlike tomato grown with S-potato onion, the overall root distribution was uniform on both sides (**Figure 1C**). In addition, compared with sole potato onion (**Figure 1D**), the root distribution of S-potato onion tended to grow towards tomato (**Figure 1E**), while the root of N-potato onion was uniformly distributed (**Figure 1F**).

### Root length density of tomato treated by root exudates from potato onion

**Figure 2:**
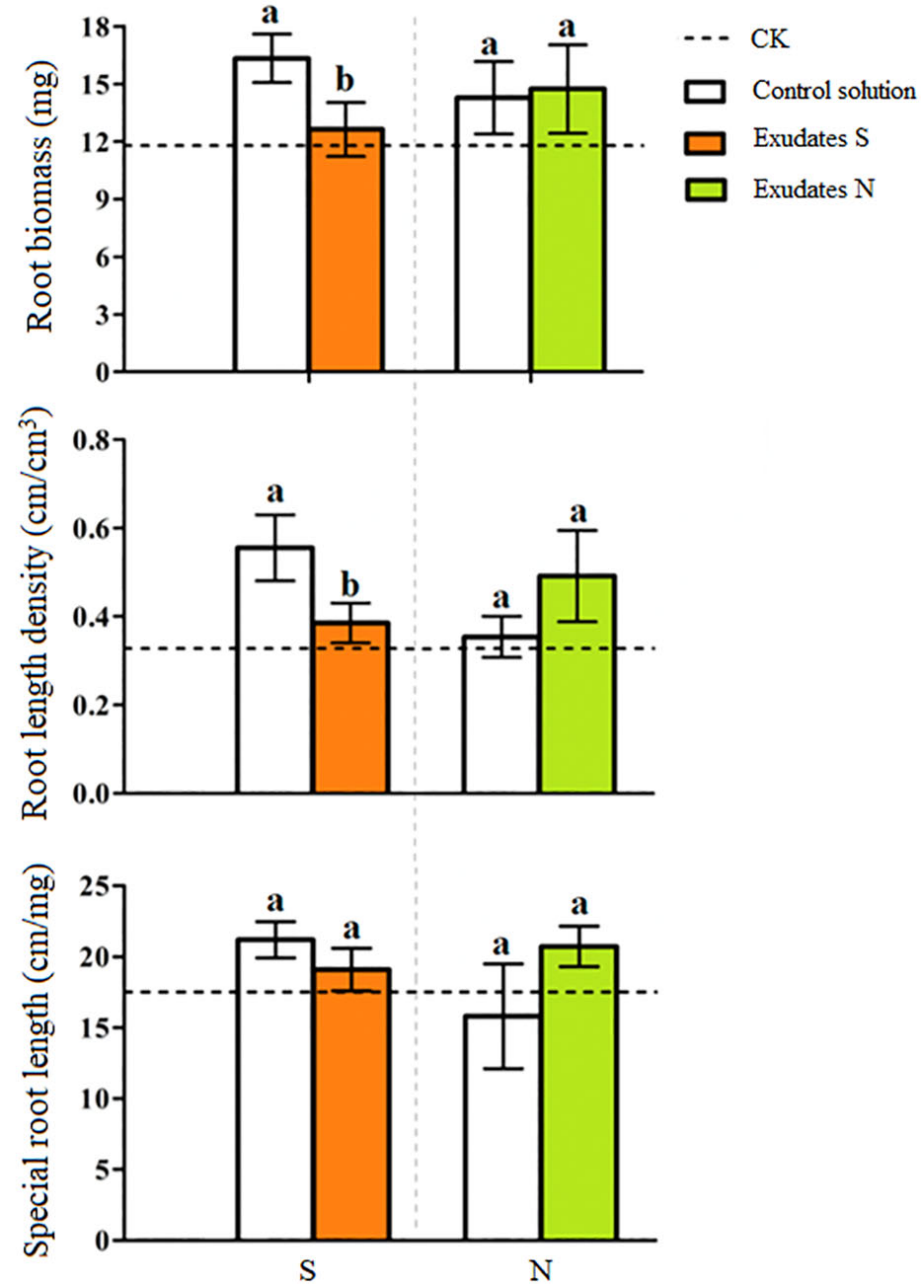
Changes in tomato root attributes treated with distilled water (CK) and root exudates of S-potato onion (S) and N potato onion (N). The dashed line is the mean value of specific tomato root attribute when no root exudates but the distilled water was applied on both sides of the tomato plant. Tomato root attributes on distilled water application side were compared with those of the root exudates application side. Values in each bar are mean ± standard error (n=4). Bars with different letters show a significant difference in root attribute based on Tukey HSD test (*P* < 0.05).

The root length density (40cm/120cm^3^ roots in upper 15 cm soil) was uniform on both sides when no potato onion root exudates were applied (**Supplementary Figure S1A**). However, as compared to control on one side, the addition of root exudates from three different potato onion cultivars or plantation types on the other side differently affected tomato root length density (**Supplementary Figures S1B–D**). The application of root exudates of sole (**Supplementary Figure S1B**) or intercropped S-potato onion (**Supplementary Figure S1C**) decreased the tomato root length density as compared to that of N-potato onion where root length density of 80cm/120cm^3^ roots was measured (**Supplementary Figure S1D**). The values of root biomass, root length density, and specific root length of tomato were greater in control than those of S-potato onion root exudates treatment, while opposite results were found when root exudates of N-potato onion were applied (*P* < 0.05) (**Figure 2**).

### UPLC-Q-TOF/MS profiling of root exudates of two potato onion cultivars

**Figure 3:**
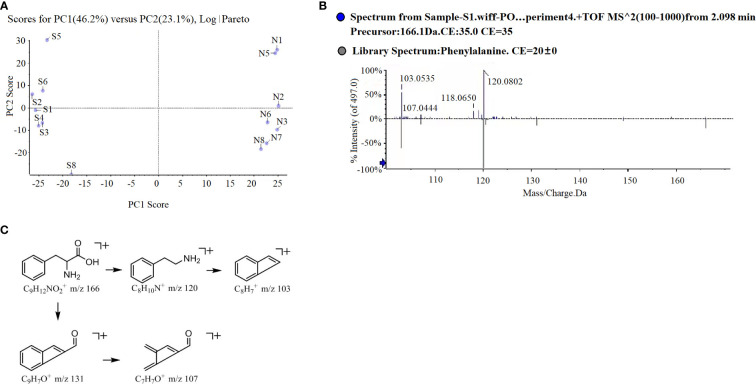
PCA analysis **(A)**, L-phenylalanine mass spectrometry **(B)** and possible cleavage pathways **(C)** of root exudates of two potato onion cultivars.

PCA analysis revealed an obvious difference in root exudate compounds of the two potato onion cultivars (**Figure 3A**). A total of 16 different prototype compounds were found in root exudates of both potato onion cultivars in UPLC-Q-TOF/MS analysis. Then, through the biological test and comparison of secondary fragments of soluble substances, we found that L-phenylalanine can alter the root growth of tomato, and the secondary fragment peaks can match the standard peak (**Figure 3B**). Next, the cleavage pathway of phenylalanine was analyzed (**Figure 3C**). The L-phenylalanine may have two cleavage pathways in the positive ion mode i.e., (i) the relative abundance peak of m/z 166[m +H]+ was strong, and the characteristic fragment m/z 120[M +H-HCOOH]^+^ was formed after the removal of HCOOH, and the characteristic fragment 103[M+H-HCOOH-NH_3_]^+^ was formed after the removal of NH3; (ii) the relative abundance peak of m/z 166[m +H]^+^ was strong, characteristic fragment m/z 131[M+H-NH_3_-H_2_O]^+^ was formed after removing NH3 and H2O, and characteristic fragment 107[M+H-NH_3_-H_2_O-C_2_]^+^ was formed after removing C2. The chemical characteristics (molecular formula: C9H12NO2, the relative molecular mass of the theoretical value: 166.08626, relative molecular mass measured value: 166.0856, error: -4%) identified the component as phenylalanine; and usually, the organisms produce phenylalanine is L, therefore considered as L-phenylalanine. Characterization of the remaining 15 different compounds were showed in **Table S1**.

### Tomato root length density and growth in response to the exogenous L-phenylalanine application

**Figure 4:**
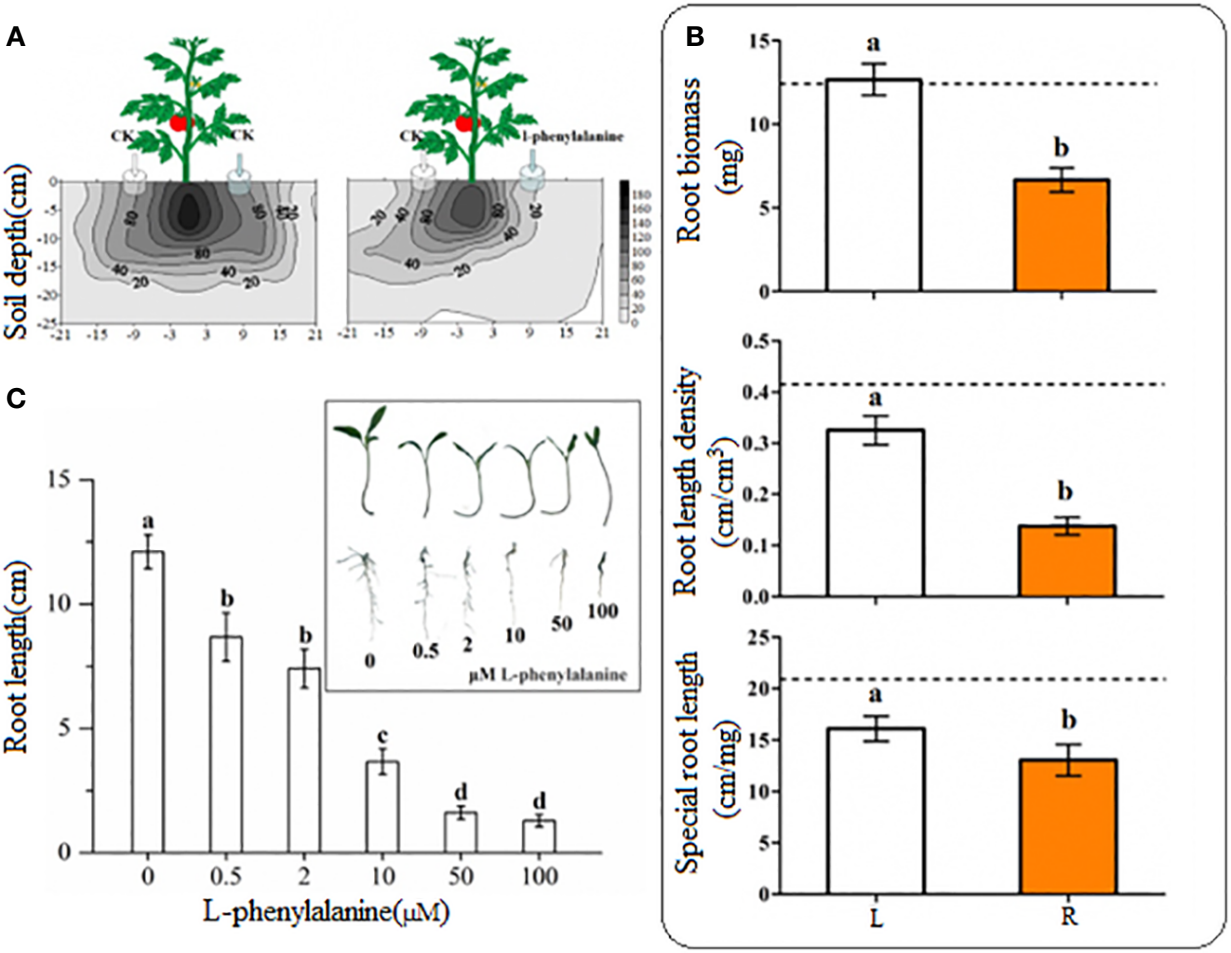
Effect of L-phenylalanine on tomato root length density distribution **(A)** and different root attributes **(B)**, and effect of different concentrations of L-phenylalanine on tomato root length **(C)**. In **(B)**, the dashed line is the mean value of specific tomato root attribute when no L-phenylalanine but the distilled water was applied on both sides of the tomato plant. Tomato root attributes in distilled water application side (L) were compared with those of the L-phenylalanine application side (R). Values in each bar are mean ± standard error (n=4). Bars with different letters show a significant difference in root attributes based on Tukey HSD test (*P* < 0.05).

Tomato roots were distributed uniformly on both sides in control (**Figure 4A**). On contrary, the tomato grew more roots toward control as compared to the side where exogenously L-phenylalanine was applied. The root length density at 20 cm/120 cm^3^ in horizontal distribution was reached 21 cm on the left side and just 10 cm on the right side and the root length density at 40 cm/120 cm^3^ in horizontal distribution reached 18 cm on the left side and 8 cm on the right side. In L-phenylalanine treatment, the treated side significantly decreased the root biomass, root length density, and special root length of tomato root as compared to the control (*P* < 0.05) (**Figure 4B**), showing that the L-phenylalanine can cause the tomato root to grow away from the addition side. All five concentrations of L-phenylalanine significantly inhibited the growth of tomato root *in vitro*, and the inhibitory effects were concentration-dependent (*P* < 0.05) (**Figure 4C**).

### Tomato root deviation angle and growth direction in response to exogenous L-phenylalanine application *in-vitro*

**Figure 5:**
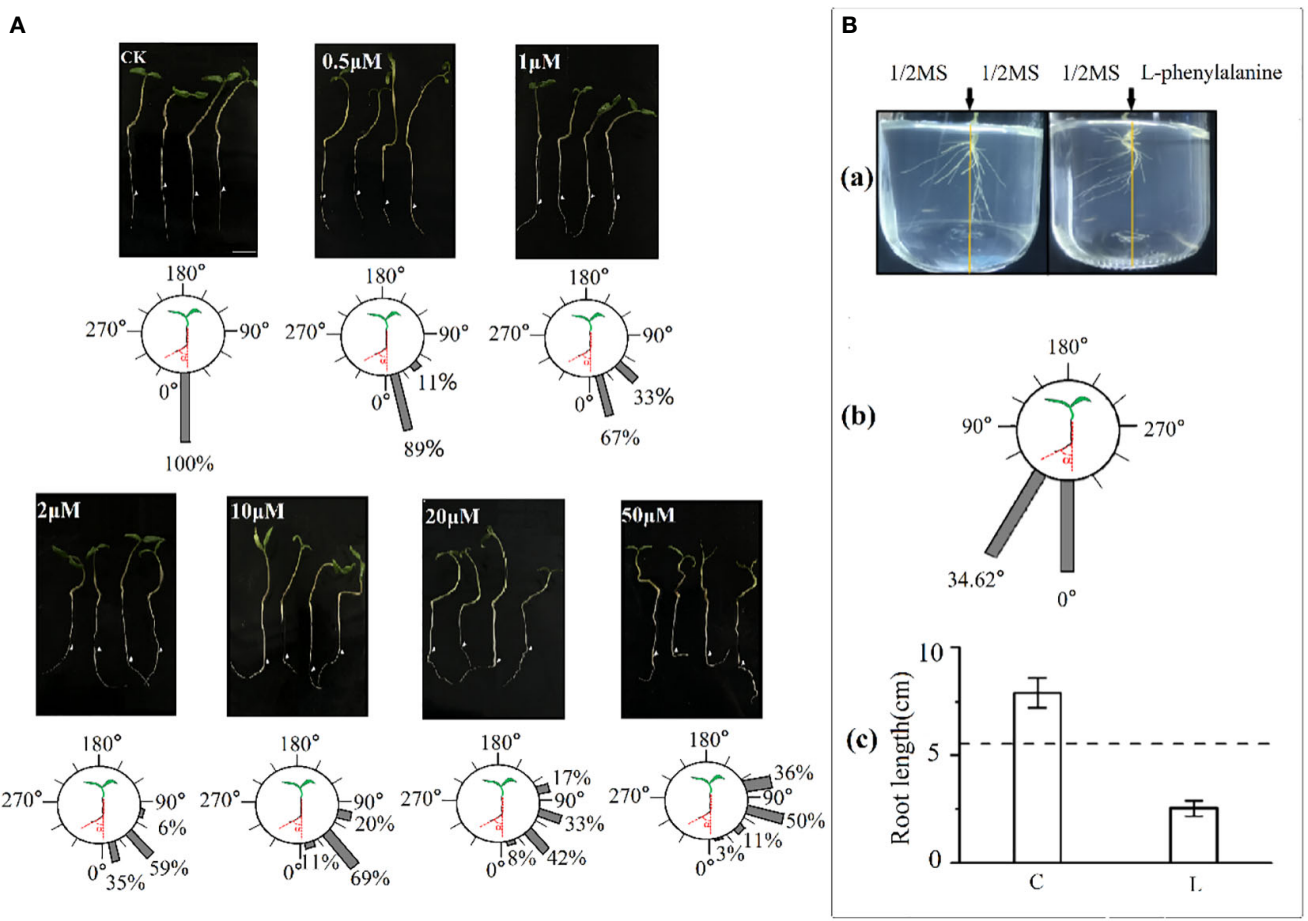
Effects of L-phenylalanine on deviation angle of tomato roots **(A)** and the root growth direction and root length of tomato **(B)**. In **B(c)**, **(C)** control (1/2MS), and L: L-phenylalanine, the difference comparison is the result of the left- and right-side in L-phenylalanine treatment, the dotted line represents the mean value of both sides in the control treatment.

Exogenous application of L-phenylalanine increased the deviation angle of tomato roots and the effects enhanced with increasing L-phenylalanine concentration (**Figure 5A**). In addition, the percentage of roots deviating angle of 0-60° was smaller and the angle of 60-120° was larger, and the deviating angle increased with increasing L-phenylalanine concentration. *In vitro* agar-culture experiment showed that tomato root grew away from the L-phenylalanine application side [**Figure 5B(a)**]. The main roots deviated from the vertical direction by 34.62° on average in L-phenylalanine treatment [**Figure 5B(b)**]. Moreover, the addition of L-phenylalanine reduced the total root length of the tomato as compared to the control (*P* < 0.05) [**Figure 5B(c)**].

### Auxin and amyloplasts distribution in columella cells of tomato roots treated with exogenous added L-phenylalanine

**Figure 6:**
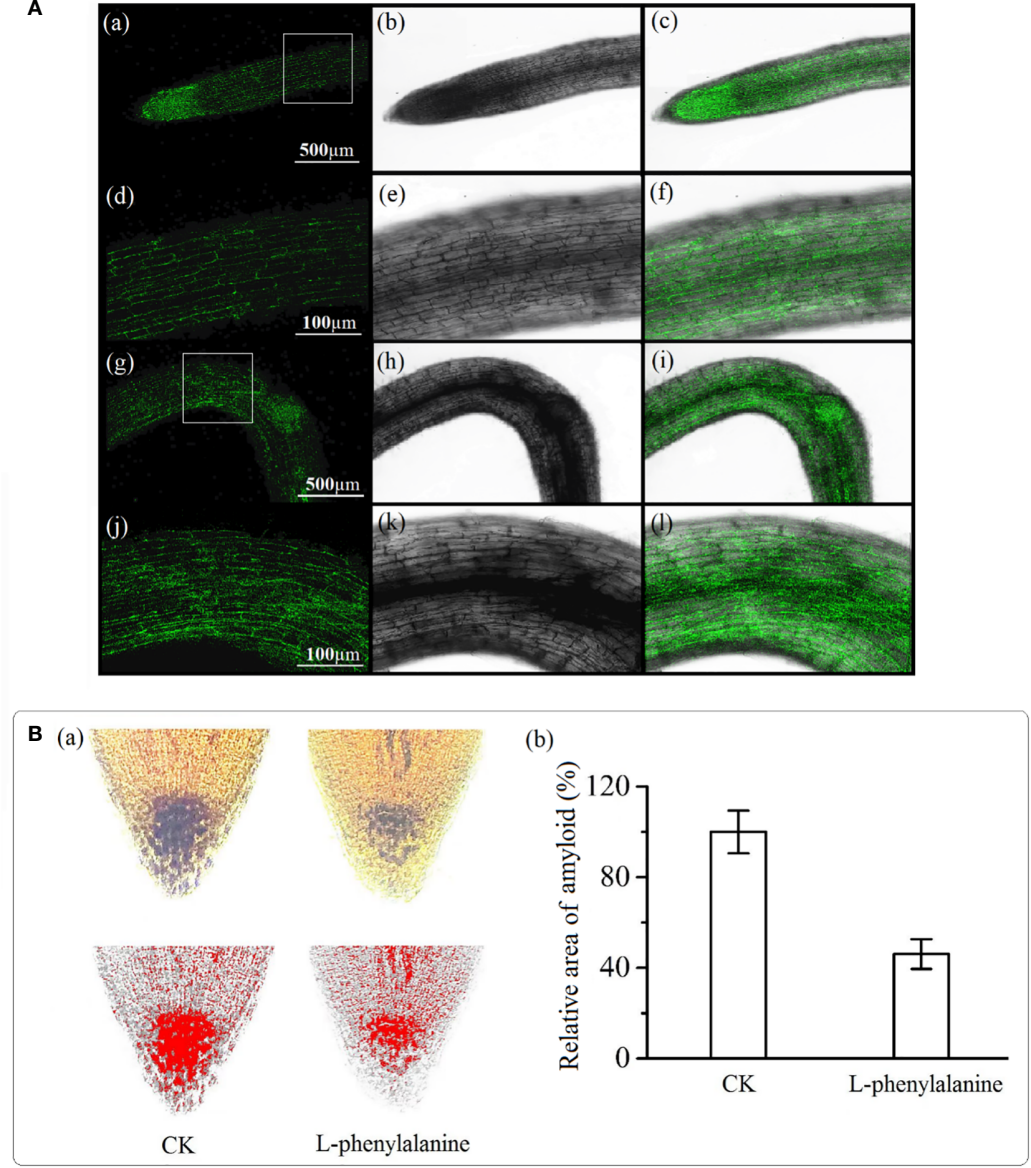
Auxin**(A)** and amyloplast**(B)** distribution in the tomato root tip. In **A**, **(a-f)**: auxin distribution in control; **(g-l)**: auxin distribution in L-phenylalanine treated roots; left column *i.e.*, **(a-j)**: auxin distribution diagram in dark field; second and third columns *i.e.*, **(b-k)** and **(c-l)**: auxin distribution diagrams in open field. In **B**, **(a)**: Image J software was used to determine the red and black area of the amyloplast; **(b)**: the amyloplast relative area in control (CK) and L-phenylalanine treated samples.

Auxin in the control group was evenly distributed in the root tip and the fluorescence intensity on both sides of the root was uniform [**Figure 6A(a-f)**]. In L-phenylalanine treatment, the fluorescence intensity of auxin on the bending side was higher than on the other side [**Figure 6A(g-l)**]. The amyloplasts were stained with iodine potassium iodide solution and visualized to examine the effect of L-phenylalanine. Compared with control, L-phenylalanine treatment significantly decreased the number of columella cells in tomato roots and reduced the amyloplast area (*P* < 0.05) (**Figure 6B**).

### The expression of genes related to the amyloplast synthesis pathway

To confirm the RNA-seq results, the expression of 15 genes which contain eight up-regulated genes, six down-regulated genes, and one gene without variation was quantified by qRT-PCR. The results of the qRT-PCR analysis were consistent with those obtained by RNA-seq analysis (**Table 1**).

**Table 1:** qRT-PCR confirmation of selected DEGs (relate to starch metabolism and auxin transport) identified by RNA-Seq.

Gene	Genes Description	Expression level	Log_2_fold-change	
			qRT-PCR	RNA-Seq
*Solyc10g078370.1*	Auxin efflux carrier component	Up-regulated	1.34	2.77
*Solyc03g118740.2*	Auxin efflux carrier component 1	Up-regulated	2.11	1.27
*Solyc07g006900.1*	Auxin efflux carrier component	Up-regulated	6.01	6.40
*Solyc05g008060.2*	Auxin efflux carrier component	Up-regulated	3.56	3.13
*Solyc01g068410.2*	Auxin efflux carrier component	Down-regulated	-2.10	-1.20
*Solyc04g007690.2*	Auxin efflux carrier component	Up-regulated	3.24	2.20
*Solyc01g111310.2*	LAX2 protein	Up-regulated	1.63	2.10
*Solyc10g076790.1*	LAX4 protein	Up-regulated	3.19	3.27
*Solyc10g055260.1*	LAX5 protein	Up-regulated	4.62	4.42
*Solyc11g013310.1*	LAX3 protein	Down-regulated	-1.46	-1.08
*Solyc01g109790.2*	Glucose-1-phosphate adenylyltransferase	Down-regulated	-2.04	-2.26
*Solyc07g019440.2*	Glucose-1-phosphate adenylyltransferase	Down-regulated	-2.38	-1.11
*Solyc07g056140.2*	Glucose-1-phosphate adenylyltransferase	Down-regulated	-1.56	-1.88
*Solyc09g091030.2*	Beta-amylase 1	Down-regulated	-2.75	-3.66
*Solyc08g083320.2*	Granule-bound starch synthase 1	Non-regulated	0.47	-0.95

Compared with control, exogenous added L-phenylalanine treatment down-regulated the expression of genes related to the amyloplast synthesis pathway, which controls the conversion of glucose 1-phosphate (G1P) to ADP-glucose, then to amylose, and finally to amyloplast. In L-phenylalanine treatment, except for the two genes solyc01g06841.2 and solyc11g01331.1, the other eight auxin transport-related genes were up-regulated. Among them, the expressions of auxin transporter carrier gene (solyc07g006900.1) and LAX5 protein gene (solyc10g055261.1) in L-phenylalanine treatment were more than 20 times higher than that in control.

## Discussion

### Root avoidance in two-species systems

Plants can identify neighboring plants and respond differently according to their identity in a diversified environment (Kong et al., 2018; Bilas et al., 2021). In the present study, we assessed the effects of two potato onion cultivars with different growth-promoting potentials on the root distribution of neighboring tomatoes. The root distribution of tomato was greatly altered in the presence of potato onion with strong growth-promoting potential, however, potato onion with weak growth-promoting potential had no obvious effect. This result indicated that changes in root trait plasticity of tomato induced by potato onion were plant species-specific.

### The role of root exudates in root avoidance

Mounting evidence shows that different plant roots avoid contact in intercropping systems, such as maize roots avoiding wheat roots (Li et al., 2006) and *Nepeta glechoma* roots avoiding strawberry roots (de Kroon, 2007). When two species co-exist, heterospecific metabolites are important cues for neighbor plant detection and subsequently trigger complex plant response strategies (Bais et al., 2006; Kong et al., 2018). In the present study, only root exudates of potato onion with growth-promoting potential changed the tomato root distribution. According to Semchenko et al. (2014), the response of plant roots to root exudates is species-specific, and there is a different composition of root exudates of different genotypes, even within the same species. Thus, the effects of different potato onion cultivars on tomato root distribution were species-specific which may be related to the differences in root exudates. Therefore, the different effects of different potato onion cultivars with growth-promoting or no growth-promoting on the tomato root distribution may be attributed to the different compositions in root exudates.

### L-phenylalanine as an “active substance” in potato onion root exudates to mediate the root-root interaction

UPLC-Q-TOF/MS analysis showed that the different substance between the two potato onion cultivars was L-phenylalanine. Exogenous application of L-phenylalanine changed the root distribution direction of tomato root, and this phenomenon was similar to the effect of tomato coculture with growth-promoting potato onion. These results indicate that L-phenylalanine is the important “active substance” that can cause the change of tomato root distribution direction in the tomato/potato onion coculture system.

As per the effectiveness of L-phenylalanine is concerned, we found that all concentrations of L-phenylalanine inhibited the growth of tomato roots and decreased root biomass and length density on the additive side. As found previously that m-tyrosine could inhibit root growth (Bertin et al., 2007; Zer et al., 2020), and L-phenylalanine can be served as a biosynthetic precursor of quinoid amino acids (Torrens-Spence et al., 2020), we speculate the inhibitory effects of L-phenylalanine on tomato roots could be due to the increase in tyrosine content in the additive side. Another possible cause is the changes in auxin concentration and distribution in the roots. Typically, a low concentration of auxin stimulates whereas a higher concentration inhibits root development (Taiz and Zieger, 2002). In our study, some auxin-related genes were highly expressed and the distribution of auxin was changed; we suppose that the root growth was regulated by auxin distribution.

In this study, L-phenylalanine inhibited the growth of tomato roots *in-vitro*, moreover, the root distribution direction of tomato was changed by both exogenously applied L-phenylalanine and tomato intercropping with S-potato onion, but the root distribution in the intercropping system was deeper. This could be because the environment of tomato roots in the coculture system was more complex than in the monoculture system, and competition among plants in diversified systems lead to changes in soil microbial diversity, nutrient distribution, and other resource availability (Xu et al., 2021; Li et al., 2022; Zhao et al., 2022). Therefore, these elements could cause tomato root expansion. For example, several soil microorganisms, such as *Trichoderma viriparum*, *Ectomycorrhizal bicolor*, and *Pseudomonas*, could promote the growth of plant lateral roots or root hairs (Contreras-Cornejo et al., 2009; Felten et al., 2009; Zamioudis et al., 2013), and changes in water and nutrients also cause root expansion (Teskey et al., 1981; Yan et al., 2022). Thus, tomato root expansion may be the result of multiple factors. We speculated that, in the coculture system, the changes in soil microorganisms, nutrients, and water caused by plant interaction induced the expansion of tomato roots to make the vertical root distribution of variable depth, meanwhile, L-phenylalanine changed the root distribution direction of tomato roots. As a result, the overall root distribution was deepened vertically and deviated to the opposite side. In future studies, the changes in soil microbial community and nutrient availability should also be considered to understand the belowground root interactions more clearly.

### L-phenylalanine-induced gravitropic responses are related to the amyloplast synthesis pathway and auxin transportation-related genes expression

Gravitation plays a key role in the formation of root architecture by affecting root growth and development, particularly in stressed environmental conditions (Cardoso et al., 2007). Many natural products can affect the response of plant roots to gravity (Santelia et al., 2008; Lupini et al., 2013). For instance, glucose (Singh et al., 2014), artemisinin (Yan et al., 2018), and hydrogen peroxide (Zhou et al., 2018) could alter the angle of plant roots deviating from the gravity direction. In this study, the angle of tomato roots deviating from the gravity direction increased with increasing L-phenylalanine concentration, indicating that L-phenylalanine changed the gravitation of tomato roots, and the agar culture experiment provided direct evidence.

The starch-filled amyloplasts in columella cells of roots perceive the gravity and deliver the directional cues to roots (Sato et al., 2015). Transcriptome analysis showed that L-phenylalanine decreased the gene expression of ADP-glucose pyrophosphorylase and β-amylase, indicating that L-phenylalanine affected the synthesis and transformation of starch in tomato roots. The staining experiment showed that L-phenylalanine decreased the number of amyloplast in tomato root tips. A decrease in the number of amyloplasts has been found in several experiments on reducing the gravitation of plant roots caused by many natural products (Singh et al., 2014; Yan et al., 2018; Zhou et al., 2018). Thus, weaker gravitation sensitivity of tomato root cells may cause by L-phenylalanine disrupting the starch synthesis in tomato roots. Therefore, the effect of L-phenylalanine on tomato root gravitropic response may result in tomato roots avoiding S-potato onion roots.

Lateral transport of auxin promotes the formation of an auxin gradient in the roots, which causes the roots to bend and grow in the direction of gravity (Santelia et al., 2008; Sato et al., 2015; Baldwin et al., 2013; Zhou et al., 2018). In the present study, L-phenylalanine stimulated the expression of genes that encode the LAX protein and PINs protein, this may have resulted in the more active transport of auxin in tomato root cells. PIN2 regulates the asymmetric distribution of auxin in the root meristem, PIN3 regulates the lateral transport of auxin and results in the different concentration distribution of auxin, and natural products i.e., artemisinin can affect the gene expression of PINs (Santelia et al., 2008; Kleine-Vehn et al., 2010; Yan et al., 2018). Thus, the effect of L-phenylalanine on gene expression of PIN2 and PIN3 may cause an asymmetric distribution of auxin in tomato roots. Laser confocal scanning microscopy analysis provided evidence that the auxin in tomato roots is mainly distributed in the cells on the medial side of the curve. The natural product affects the gravitation of roots by abnormal horizontal redistribution of auxin (Yan et al., 2018). This might be because L-phenylalanine up-regulated the auxin transportation-related genes, resulting in the asymmetric distribution of auxin in tomato roots in our study. Although we have found L-phenylalanine in S-potato onion root exudates changing the distribution and growth direction of tomato roots. However, some other substances may also play important role in changing tomato root distribution, which needs further research. Moreover, most substances in root exudates have synergistic/composite effects, thus the synthesis of substances in root exudates needs to be further explored. Besides, the changes in rhizosphere microbial community composition (Lange et al., 2015; Zhong et al., 2020) and nutrients availability (u Rahman et al., 2021) by the cocultured heterospecific plant may trigger different physio-morphological responses in the focal plant. Therefore, the combined effect of root exudates, soil microorganisms, and nutrients on plant root distribution during interspecific interaction would be exciting to explore in the future.

## Conclusions

In the current study, we demonstrated that L-Phenylalanine, derived from the growth-promoting potato onion cultivar, could be the important “active substance” that causes the change of tomato root distribution direction in the tomato/potato onion coculture system. The involved mechanism could be that L-Phenylalanine changed the auxin distribution of roots and decreased the concentration of amyloplasts in columella cells, then altered the root deviation angle. Our findings provide strong evidence that the chemical communication of root exudates occurs in the process of root-root interaction. These ecological observations provide another aspect of plant-plant communication and the critical role of root exudates in plant ecological interactions. However, the influence of L-phenylalanine on tomato root cannot fully represent the root interaction result in the coculture system; therefore, the effects of other substances in root exudates, soil microorganisms, and nutrient availability on root distribution need to be comprehensively considered in future studies.

## Materials and methods

### Plant material and soils

Tomato (cv. Dongnong 708) seeds were obtained from Tomato Breeding Center, Northeast Agriculture University, Harbin, China. The bulbs of potato onion cultivars Suihua (S-potato onion) and Ningan Hongcheng (N-potato onion) were obtained from the Laboratory of Vegetables Physiological Ecology of the Northeast Agricultural University. The soil used was sandy loam soil collected from the soil upper layer (0-15 cm) of an open field in Horticulture Experimental Station, Northeast Agricultural University. The soil physicochemical properties were: organic matter, 22.32 mg kg^-1^; inorganic nitrogen, 86.95 mg kg^-1^; available phosphorous, 37.23 mg kg^-1^; available potassium, 102.42 mg kg^-1^; electrical conductivity (1:2.5, w/v), 163.38 µS cm^-1^; and pH (1:2.5, w/v), 6.68.

### Experimental layout

#### Experiment 1: Tomato root length distribution in coculture experiment

**Figure 7:**
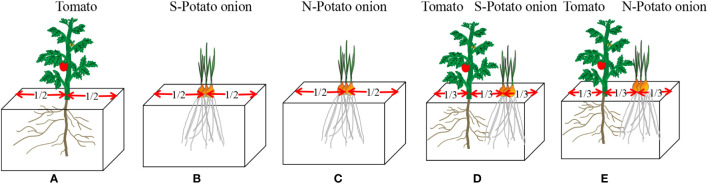
Schematic diagram of five types of plantations. **(A)** treatment (i) tomato monoculture; **(B)** treatment (ii) S-potato onion monoculture;**(C)** treatment (iii) N-potato onion monoculture;**(D)** treatment(iv) tomato cocultured withS-potato onion; **(E)** treatment (v) tomato cocultured withN-potato onion.

Polythene foam boxes (1, 36 × 25 × 22cm) containing 20 kg of soil each were used. At two cotyledons, tomato seedlings were transferred to experimental boxes (one seedling per box), and simultaneously potato onions were planted (three bulbs per box). There were five types of plantations, i.e., (i) tomato monoculture, (ii) S-potato onion monoculture (iii) N-potato onion monoculture (since the root distribution result of N-potato onion monoculture is very similar to S, the result of N-potato onion monoculture was not list), and tomato cocultured with (iv) S-potato onion and (v) N-potato onion (**Figure 7**). Each type of plantation had four boxes and was repeated three times. Boxes were placed randomly without any order and their placement was randomized once a week. Water was applied regularly to maintain 55 ± 5% of the water holding capacity. No fertilizers were applied and weeds were uprooted manually. After 20 days of tomato transplantation, tomato seedling root length density was measured.

Root length density was measured by the Monolith method (Li et al., 2006; Wang et al., 2014; Yu et al., 2017). The experimental boxes were cut horizontally and soil samples of the middle 5 cm thick soil layer were taken up to 20 cm of soil depth using an iron box (6 cm × 5 cm × 4 cm), and numbered according to the section sequence (six pieces horizontally and five pieces vertically; 30 pieces in total per box). Since the roots mixed together in the companion cropping system, the tomato and potato onion roots were distinguished by differences in color, smell, and fiber characteristics. For example, tomato roots are yellowish and hairy, whereas potato onion roots have a smooth surface with white coloration and some degree of transparency. The separated root fractions were then scanned with an image scanner analyzer (LA-S2400), yielding data from each soil core in terms of total root length. Root length density was calculated by combining root length and soil volume.

#### Experiment 2: Collection of root exudates and identification of “active substances”

Three plantation types, i.e., (i) S-potato onion monoculture, (ii) N-potato onion monoculture and (iii) S-potato onion cocultured with tomato (TS-potato onion, our previous study have proved the S potato onion has growth-promoting effect in tomato/potato onion coculture system, in order to exclude the effect of S root exudates is due to concomitant, this treatment was set. N root exudates mainly plays a contrast role, so the “TN” treatment was not set.), were set up and raised as described above in intercropping experiment section. Twenty days later, root exudates of potato onion were collected separately following the methods previously used by Li et al. (2013). One part of the collected root exudates was used in box experiment (2). The other root exudates were concentrated by freeze-dry and then used for UPLC-Q-TOF/MS analysis.

#### Experiment 3: Tomato root length distribution responses to potato onion root exudates

Tomato seedlings with two cotyledons were transplanted in the center of the experimental box (2). Three days after transplantation, 25 ml of collected potato onion root exudates were poured using a fixed plastic pipe (5 cm length, 5 cm diameter) 10 cm right to the tomato seedling, while the same volume of distilled water was poured 10 cm left to tomato seedling (control) with a three days interval (**Figure 2**). The amount of treatment solution increased gradually each week with the growth of the plant (i.e., 25ml→30ml→35ml) as previously suggested by Semchenko et al. (2014). There were four treatments i.e., (i) distilled water was poured in both sides, and distilled water was poured on the left side while root exudates of (ii) S-potato onion, (iii) N-potato onion, and (iv) S-potato onion cocultured with tomato were poured in right side. Each treatment was tested in four boxes which were further repeated three times. After 20 days, root length distribution was measured.

#### Experiment 4: *In vitro* assessment of L-phenylalanine efficiency on tomato roots

##### Box experiment to test the tomato root distribution affected by L-phenylalanine

Tomato seedlings with two cotyledons were transplanted in the center of the experimental box (3). Three days after transplantation, L-phenylalanine solution (0.829 µM) was applied to the soil using the same method as used for exogenous application of root exudates treatments. Previously, we have found that the root mass density of tomato was 2-22 g fresh weight/kg soil (Yu et al., 2017), and we choose the average (10g fresh weight/kg) to measure the L-phenylalanine which was 0.478 µg/g fresh weight of root. Therefore, we assumed that the L-phenylalanine weight was 6.837 µg in 0.65 kg of soil, thereby the used concentration was calculated as 0.829 µM. For control, only distilled water was poured on both sides, whereas for L-phenylalanine treatment, distilled water was poured on one side while L-phenylalanine solution was poured on the other side (**Figure 3A**). Each treatment was tested in four boxes which were further repeated thrice. After 20 days, root length density, total root length, and root dry biomass were measured as described above.

##### Petri dish experiment to test the tomato growth affected by L-phenylalanine

Tomato seedlings with roots about 1 cm were selected for the experiment after taking off the seed coat. Two layers of qualitative filter paper were placed into the petri dish, and 5 ml of control or L-phenylalanine solution was added. Five concentrations of L-phenylalanine were: 0.5, 2, 10, 50, and 100 µM. There were six treatments, each treatment was repeated four times with five tomato seedlings per replicate. The total root length was measured as described above.

##### Plate tissue culture experiment to test the tomato root deviation angle responses to exogenously applied L-phenylalanine

The tomato seeds were disinfected with 5% sodium hypochlorite for 10 s, rinsed with 70% anhydrous ethanol for 30 s twice, and then rinsed in sterile water 5-6 times. After disinfection, seeds were placed on the conventional medium in petri dish (25 ml medium per petri dish). The conventional medium was prepared as 10 g sucrose and 5 g plant gel, and 2.46g 1/2MS medium was added into 800 ml ultrapure water. The pH was adjusted to 5.8 and the constant volume was adjusted to 1000 ml. After sterilization at 120°C for 20 min, the medium was cooled to about 60°C and pour 25 ml medium into each petri dish. The petri dishes were placed vertically to ensure that the seed radicle was vertically downward and placed in a light incubator with 28/18°Ctemperature and 60% humidity. After 7 days of culture, the roots with the same root length were transferred to the treated medium and sampled after 48 h.

The final concentrations of 0 μM (control), 0.5 μM, 1 μM, 2 μM, 10 μM, 20 μM and 50 μM were obtained by mix different concentrations of L-phenylalanine into conventional medium.

About 150 ml of the medium was poured into each petri dish (diameter 15 cm), and cooled to room temperature. Each treatment was repeated thrice, 4 tomato seedlings with the same root length were placed in the upper third of the petri dish, and the petri dishes were placed vertically to ensure that the seed radicle was vertically downward and placed in a light incubator with 28/18°Ctemperature and 60% humidity. Each replicate contained 12 tomato seedlings in total. Samples were collected after 48 h, and the photos of the roots were taken to determine the root deviation angle.

##### Culture experiment in culture flask to test the degree of tomato taproot deviation from vertical direction and root length affected by L-phenylalanine

Tomato seedlings were germinated as above. At about 5 mm root length, seedlings were transferred to a culture bottle (7 cm × 11 cm). The conventional medium was prepared as 10 g sucrose and 5 g plant gel, and 2.46g 1/2MS medium was added into 800 ml ultrapure water. The pH was adjusted to 5.8 and the constant volume was adjusted to 1000 ml. After sterilization at 120 °C for 20 min, the medium was cooled to about 60 °C. The culture medium was divided into two parts; one half was conventional medium (non-L-phenylalanine) and the other half was supplemented with 0.829 µM L-phenylalanine. Two treatments were set up in the experiment, i.e., control and L-phenylalanine. Each treatment was repeated three times and four tomato seedlings were used for each replicate. The root growth response was monitored visually and photographed, then the angle of taproot deviated from vertical direction was measured with Image J software, the root length was measured as above.

### Experimental methodology

#### Collection of root exudates

Root exudates of potato onion from three plantation types were collected separately following the methods previously used by Li et al. (2013). Briefly, After 20 days of cultivation, roots of potato onion in different treatments were gently collected from soils and washed with tap water, then washed with autoclaved water. Cleaned roots were completely submerged in a beaker with 200 mL of autoclaved deionized water, and were placed in a plant growth chamber for 6 h at 24°C with light. To maintain the osmotic pressure of roots, 0.5 mM CaCl_2_ was added (Vranova et al., 2013). During the collections, each beaker contained 5 seedlings and covered by tinfoil to avoid contamination and light. Refill the beaker to 200 ml with deionized water every 2h during this period. Roots were dried using absorbent paper, and weighed to adjust the concentration of aqueous solution to 1g fresh weight of 10 ml solution, finally. The final solution was filtered through a 0.22 µm millipore filter and stored at –80 °C.

#### Identification of the different substances between N- and S- potato onion root exudates

##### UPLC-Q-TOF/MS analysis

Freeze-dried powder of potato onion root exudates were dissolved with methyl alcohol, extracted in an ultrasonic bath for 30 min at 30°C. The extracts were filtered through a 0.22 µm membrane. A Waters Acquity UPLC system (Waters Corp., MA, USA) with BEH C18 column (2.1 mm × 100 mm, 107 µm) and BEH C18 VanGuard pre-column (2.1 mm × 5 mm, 1.7 µm) was used for chromatographic separation of the samples. The temperature of the column was set and maintained at 35 °C. Ultrapure water (A) and methyl alcohol (B) were used to make the mobile phase. The separation was obtained by employing a binary gradient elution (flow rate, 0.3 ml min-1) using a consecutive program as: 0.3 ml/min, 0–5.0 min, 95%~60% A; 5.0–15.0 min, 60%~0 A; 15.0–22.0 min, 0% A; 22.0–22.1 min, 0%–95% A, 22.1–26.0 min, 95% A. The temperature of the sample bin was adjusted to 4 °C. The injection volume of the sample was 2 µL. The mobile phase, after chromatographic separation, was introduced into a hybrid triple Q-TOF mass spectrometer [TripleTOF 5600+ (AB SCIEX, Singapore)] equipped with a TurboIonSpray and the Turbo V sources. The MS condition used were as follows: ESI+/ESI–mode; ion spray voltage of 5.5/-4.5 kV; collision energy of 40/-40 eV; collision energy spread of 20/-20 eV; nebulizer gas pressure of 55psi; heater gas pressure of 55 psi; and curtain gas pressure of 35 psi. Nitrogen was used as auxiliary gas and nebulizer and the scans of TOF-MS were run in a mass range of 80–1600 m/z. The eight most intense fragment ions of each analyte with >100 counts s-1 were screened for a production scan of 50–1500 m/z. The MS and MS/MS were regulated by an automated calibration delivery system. The Analyst TF 1.6 (AB SCIEX, CA, USA) was used to obtain the data, which was analyzed using MarkerView 1.2.1, MasterView 1.0 and PeakView 2.0 (AB SCIEX, CA, USA).

##### Non-targeted peaks identification and components analysis

The peaks were matched and picked using MarkerView 1.2.1, and the data processing was performed as follows: retention time, 0.5–30.0 min; retention time tolerance, 0.2 min; peak intensity threshold, 100; mass tolerance, 0.02 Da; maximum elements, C50H100O30; maximum tolerance, 10 ppm; and peak area, 3000 ions. The noise was eliminated automatically. The isotopic peaks, but not the specific mass or adduct, were excluded. The data model consisting of a score plot, showing the principal component analysis (PCA) scores, and a loading plot, showing PCA loadings for ions and comparisons of PC1 and PC2, was created. Ions were represented as markers. Markers far from the center were considered the leading contributors differentiated between fresh and dry samples.

##### Identification of material structure

Firstly, the molecular weight of the substance with an error range of 5×10^-6^ was analyzed by the Triple TOF TM5600 system according to the second-order ion fragments, and the isotope abundance ratio was determined. Secondly, the Formula Finder function was used to determine the compound formula by combining the molecular weight of the substance and the isotope abundance ratio (isomers cannot be distinguished). Then, fragment composition was inferred based on the mass number of fragment ions and the possible structural formula was obtained from the ChemSpider database. The decomposition characteristics of the compound were analyzed in Peakview 2.0, and the structure of the compound was inferred by combining with the similarity degree of the secondary spectrogram. Finally, the compound with standard substance among the different substances was compared and identified according to the retention time of standard substance and secondary mass spectrometry to further determine the material structure.

#### Observation and measurement of amyloplasts in the root columella cells

About 1 mm length was taken from the root tip of the tomato and invaded in a mixed solution [0.4%(w/v) acetic acid, 45% (w/v) anhydrous ethanol] at 4°C for 48 h. Then, the root tip was taken out with a light clamp and placed on a slide, stained with I2-KI solution at room temperature for 8 min, rinsed with double distilled water once and excess dye was absorbed. The film was lightly pressed. The Image J software was used to record the amyloplast area.

#### Immunofluorescent tissue localization of auxin

The immunofluorescent staining technique was used to detect the IAA distribution in the root tip elongation zone. Briefly, 500 µm of 1 N of NaOH was added to 900 ml double distilled water and stirred in 40 g of paraformaldehyde at 60°C to prepare a paraformaldehyde stationary liquid. After cooling down to room temperature, 100 ml of TBS (10x) buffer was added and pH was adjusted to 7.4 with HCl (Schlicht et al., 2006). The staining was done using Immunol Fluorescence Staining Kit (KeyGEN BioTECH, Nanjing, China) according to the manufacturer’s protocol. Seedlings treated with or without H_2_O_2_ for 60 h were fixed with 4% paraformaldehyde stationary liquid at 4°C for 1 h. Then, an ice-cold PBS buffer solution was used to wash the samples three times each. Samples were submerged in diluted monoclonal antibody anti-IAA (diluted 1:20 in PBS supplemented with 1% Bovine Serum Albumin buffer) and incubated in a humidity box at 4°C overnight. Seedlings were then washed with PBS thrice and incubated in secondary antibody FITC–labeled goad anti-mouse IgG 1% BSA in dark at room temperature for 1 h. After that, seedlings were taken out and washed with ice-cold PBS in the dark, and observed immediately using a fluorescence confocal microscope (excitation, 490 nm; emission, 520 nm; Olympus FV 1000 MPE).

#### Analysis of differently expressed genes in tomato roots

In L-phenylalanine treatment, the lateral fine roots of the L-phenylalanine addition side were collected, while in control, the fine root was randomly selected on both sides. For each treatment, three samples were prepared, where each sample was a pooled sample of roots from three seedlings. The samples were named CK1, CK2, CK3, L-Phe1, L-Phe2, and L-Phe3. All the samples were frozen in liquid nitrogen, and stored at –80°C before transcriptome sequencing analysis which was employed at Majorbio Bio-pharm Technology Co., Ltd., Shanghai, China, according to their standard protocol (http://www.illumina.com/). Acquisition of the clean reads, map to tomato genome reference, transcript assembly, and determination of differently expressed genes were as described by Fu et al. (2015).

#### Quantitative PCR analysis for validation of RNA-seq results

Randomly selected 15 genes associated with amyloid synthesis and auxin transport were analyzed in qRT-PCR for validation of RNA-seq results. The Primer 5.0 software was used to design the gene-specific primers, that were synthesized by Sangon Biotech Company (Shanghai, China). The genes and primer are listed in **Table 2**. The samples used for qRT-PCR were the same as used for RNA-seq analysis, the synthesis of cDNA, and the reaction condition of qRT-PCR as described by Fu et al. (2016).

**Table 2:** Genes and priers used in real-time qRT-PCR analysis.

Gene	Forward primer	Reverse primer
*Solyc10g078370.1F*	5’-CAGTCGGACTTCGAGGTGTC-3’	5’-GAGGCCTCACCCTGTGCTC-3’
*Solyc03g118740.2F*	5’-AGGTCTGTTCATGGCTTTGC-3’	5’-GTAGTAGGTCATAGTGTAGAGATTG-3’
*Solyc07g006900.1F*	5’-CGATTGGTCTTCGCGGTGTA-3’	5’-ACGAACCAGAAGCAGATTCAAA-3’
*Solyc05g008060.2F*	5’-AATGTTGCATTAAAATTGTTGGTTC-3’	5’-TGGCAACGCTATCAACATCC-3’
*Solyc01g068410.2F*	5’-TTATCCCTTAAGATCCGGCCA-3’	5’-TCTTGTTTGAAATTGAGAGGTCACT-3’
*Solyc04g007690.2F*	5’-GTGGGAACACTGTGGCTACT-3’	5’-TGCATTGGCCTAATACATCTCT-3’
*Solyc01g111310.2F*	5’-CCACCATTCCTCCCTCATGT-3’	5’-TAAGCAAAACTCAAGACGTGGG-3’
*Solyc10g076790.1F*	5’-TGCTCGACAGGTATTTCCAT-3’	5’-ATTCCTCATTTTCACGCGCT-3’
*Solyc10g055260.1F*	5’-AATCCCTGCACTTGCTCACA-3’	5’-TGGAGTTGAAGAAATGACATCCCA-3’
*Solyc11g013310.1F*	5’-AGAGAGGTAACCCTAGCGAA-3’	5’-TTGACCATACTTGCCCACCC-3’
*Solyc01g109790.2F*	5’-TTGACAAGAACGCAAAGATAGG-3’	5’-AGTGCTCCCATGCTACAAACA-3’
*Solyc07g019440.2F*	5’-AACTGAGCTCTGGTCCTCTGA-3’	5’-CAGCTTCTTCAACACCCTGC-3’
*Solyc07g056140.2F*	5’-TGGTTTACTGCGGAGCAACT-3’	5’-TGCATGTTCAAATGACACTAAGTC-3’
*Solyc09g091030.2F*	5’-ACAAGAAGCTGCAGTAGCCC-3’	5’-ACGAACGAACATCAACCAGTC-3’
*Solyc08g083320.2F*	5’-TGCGATGTTGTTGACCCAGA-3’	5’-CAGTTGGCATCCATCAGTGC-3’
Actin	5’-TGAATGCACGGTAGCAAACAACAGATT-3’	5’-AATGCATCAGGCACCTCTCAAGTAT-3’

### Statistical analysis

Data obtained from the monoliths of the experimental boxes represent the entire root population in each soil profile. The results are presented in contour diagrams which were prepared using the Surfer 8.0 software (Holden Software Inc., CO, USA). The scanning of roots was performed by MICROTEK (Scan Maker i800 plus) and obtained results were analyzed by the plant root analysis system WS (LA-S2400). Data were analyzed by ANOVA and the mean values of groups were compared based on the Tukey HSD test at a significance level of *P* < 0.05. The graphical visualization of results was performed by Origin 8.5. Data were expressed as mean ± standard error. The material structure was derived by Chemsdraw. Material structure analysis and UPLC-Q-TOF-MS/MS condition optimization were completed with the assistance of the Heilongjiang National Institute of Medicine, Harbin, China.

## Data availability statement

The original contributions presented in the study are publicly available. This data can be found here: NCBI, SRP254411.

## Author contributions

FW conceptualized, designed, obtained the funding, and supervised the work. HY, SC and DG conducted the experiment, HY, SC, DG and MK analyzed the data and wrote the manuscript. All authors reviewed and approved the final manuscript.
