# Supplementary material for: L-phenylalanine in potato onion (Allium cepa var. aggregatum G. Don) root exudates mediates neighbor detection and trigger physio-morphological root responses of tomato
Source: Front Plant Sci. 2023 Feb 16;14:1056629. doi: 10.3389/fpls.2023.1056629 (PMC9981155; doi:10.3389/fpls.2023.1056629)
Supplement: Supplementary file 1 [file DataSheet_1.pdf]

L-phenylalanine from potato onion root exudates mediate neighbour detection and trigger physio-morphological root responses in adjacent tomato

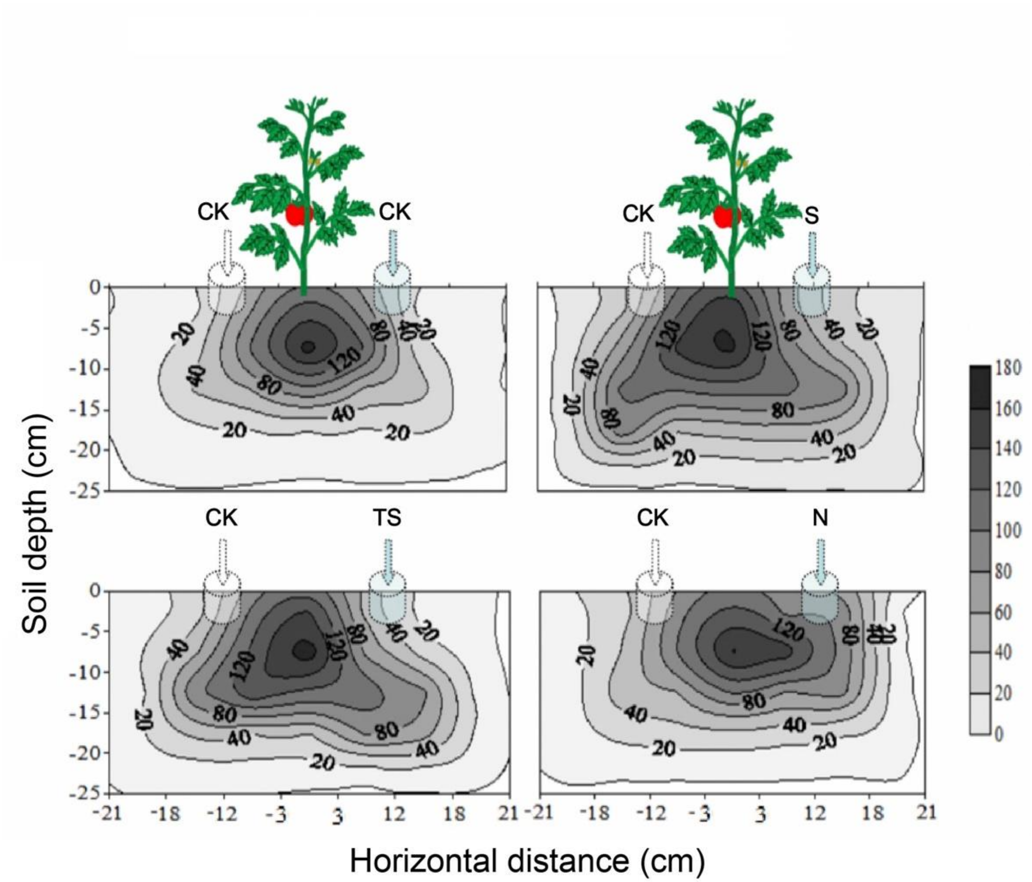

Fig. S1 Effect of distilled water (CK) or root exudates of S-potato onion (S), N-potato onion (N) and tomato intercropped S-potato onion (TS) on tomato root distribution. The scale represents the special root length density, and the shading density indicates the root intensity.

Table S1 Characterization of the remaining 15 different compounds by UPLC-ESI-Q-TOF/MS

Num.	Retention time(min)	Iron modle	Measured mass	Calc mass	Error (10^{-6})	Peak heights (Response value)		Formula	MS/MS fragmention	Compounds
						N	S			
1	7.2	$[M+H]^+$	212.0195	212.01896	2.5	—	315099.62	$C_8H_5NO_6$	212[M+H]+,194[M+H-H2O]+,168[M+H-H2O-C2H2]+,166[M+H-H2O-C2H2-H2]+	2,4,6-Pyridinetricarboxylic acid
2	14.4	$[M+H]^+$	247.2037	247.20564	-7.8	523646.50	—	$C_{17}H_{26}O$	247[M+H]+,229[M+H-H2O]+,219[M+H-C2H4]+,203[M+H-C2H4-O]+,189[M+H-C2H4-O-CH2]+,177[M+H-C2H4-C3H6]+,124[M+H-C2H4-C3H6-C4H5]+,105[M+H-C2H4-C3H6-C4H5-H3O]+,	4-(trans-4-Pentylcyclohexyl)phenol
3	7.9	$[M+H]^+$	416.3515	416.35231	-1.9	—	95854.70	$C_{27}H_{45}NO_2$	416[M+H]+,398[M+H-H2O]+,273[M+H-H2O-C8H15N]+,255[M+H-2H2O-C8H15N]+,161[M+H-2H2O-C15H25NO2]+	Cevane-3,6-diol
4	12.1	$[M+H]^+$	291.1939	291.19547	-5.4	456257.000	—	$C_{18}H_{26}O_3$	291[M+H]+,273[M+H-H2O]+,273[M+H-H2O]+,175[M+H-H2O-C5H6O2]+,133[M+H-H2O-C5H6O2-C3H6]+,231[M+H-H2O-C2H2O]+,213[M+H-H2O-C2H2O-H2O]+,157[M+H-H2O-C2H2O-H2O-C4H8]+,	4-(4-Octylphenyl)-4-oxobutanoic acid
5	13.0	$[M+H]^+$	252.1586	252.15942	-3.3	—	210291.20	$C_{14}H_{21}NO_3$	252[M+H]+,235[M+H-OH]+,220[M+H-OH-CH3]+,207[M+H-OH-CHNH]+,190[M+H-OH-CHNH-OH]+,175[M+H-OH-CHNH-OH-CH3]+,163[M+H-OH-CHNH-OH-CH3-C]+,149[M+H-OH-CHNH-OH-CH3-C-CH2]+,	2-Amino-2-oxoethyl adamantan-1-ylacetate
6	7.7	$[M+H]^+$	297.2904	297.29004	1.2	—	112340.180	$C_{18}H_{36}N_2O$	297[M+H]+,238[M+H-C3H9N]+,167[M+H-C3H9N-C4H9N]+,	1-(4-Ethyl-1-piperazinyl)-1-dodecanone
7	1.7	$[M+H]^+$	311.1182	311.11789	1.0	—	144572.50	$C_{21}H_{14}N_2O$	311[M+H]+,251[M+H-CH4N2O]+,239[M+H-CH4N2O-C]+,222[M+H-C7H5]+,210[M+H-C7H5-C]+,179[M+H-C7H5-C2H3O]+,	6,7,12,13-Tetrahydro-5H-indeno[2,1-a]pyrrolo[3,4-c]carbazol-5-one
8	12.2	$[M+H]^+$	319.1879	319.1877	0.6	51124.380	—	$C_{15}H_{22}N_6O_2$	319[M+H]+,258[M+H-H3N3O]+,243[M+H-H3N3O-CH3]+,181[M+H-C7H12N3]+	N-(2-Methoxyethyl)-1-(3-methyl[1,2,4]triazolo[4,3-b]pyridazin-6-yl)-4-piperidinecarboxamide
9	16.7	$[M+H]^+$	323.2576	323.25807	-1.5	—	487727.97	$C_{20}H_{34}O_3$	323[M+H]+,291[M+H-CH4O]+,235[M+H-CH4O-C4H8]+,193[M+H-CH4O-C4H8-C2H2O]+,165[M+H-CH4O-C4H8-C2H2O-C2H4]+,	(3aS,5S,6aS)-5,6a-Diethyl-5-[(5E)-4-ethyl-5-octen-1-yl]tetrahydrofuro[3,2-b]furan-2(3H)-one
10	12.4	$[M+H]^+$	360.2165	360.21694	-1.2	276219.12	—	$C_{21}H_{29}NO_4$	360[M+H]+,342[M+H-H2O]+,175[M+H-H2O-C8H9NO3]+,119[M+H-H2O-C8H9NO3-C4H8]+,332[M+H-CO]+,332[M+H-CO]+,332[M+H-CO]+,156[M+H-CO-C13H20]+,	{2-[(4aR,6R)-2,3,4,4a,6,6a,7,8,9,10b-Decahydro-1H-benzo[c]chromen-6-yl]phenoxy}acetic acid ammoniate
11	14.3	$[M+H]^+$	383.278	383.28054	-6.6	247126.19	—	$C_{23}H_{34}N_4O$	383[M+H]+,291[M+H-C6H6N]+,235[M+H-C6H6N-C4H8]+,165[M+H-C6H6N-C4H8-C4H6O]+,109[M+H-C6H6N-C4H8-C4H6O-C4H8]+,	1-(1-Azepanyl)-3-{1-[(4-methyl-1-piperazinyl)methyl]-1H-indol-3-yl}-1-propanone
12	15.7	$[M+H]^+$	431.2214	431.22169	-0.7	99199.173	—	$C_{28}H_{30}O_4$	431[M+H]+,319[M+H-C8H16]+,303[M+H-C8H16-O]+,241[M+H-C14H22]+,163[M+H-C14H22-C6H6]+,	Bis(4-butylphenyl) isophthalate
13	20.2	$[M+H]^+$	439.3235	439.32067	6.4	909888.42	—	$C_{29}H_{42}O_3$	439[M+H]+,327[M+H-C8H16]+,249[M+H-C8H16-C6H6]+,215[M+H-C8H16-C8H16]+,137[M+H-C8H16-C6H6-C8H16]+	Benzophenone, 4-hexadecyloxy-2-hydroxy

Num.	Retention time(min)	Iron modle	Measured mass	Calc mass	Error (10^{-6})	Peak heights (Response value)		Formula	MS/MS fragmention	Compounds
						N	S			
14	15.1	[M+H]+	637.3072	637.3066	0.9	272757.96	—	C29H48O15	637[M+H]+,581[M+H-C4H8]+,525[M+H-C4H8-C4H8]+,469[M+H-C4H8-C4H8-C4H8]+,393[M+H-C4H8-C4H8-C4H8-C6H4]+,337[M+H-C4H8-C4H8-C4H8-C6H4-C4H8]+,	Methyl 6-O-[2,3,4-tris-O-(2,2-dimethylpropanoyl)-6-methyl-β-D-glucopyranuronosyl]-β-D-galactopyranoside
15	20.1	[M+H]+	663.4557	663.45521	0.7	1037125.40	—	C33H58N8O6	663[M+H]+,607[M+H-C4H8]+,551[M+H-C4H8-C4H8]+,495[M+H-C4H8-C4H8-C4H8]+,439[M+H-C4H8-C4H8-C4H8-C4H8]+	N-(1-Oxidoisonicotinoyl)-L-isoleucyl-N-[(2S)-1-{[(2S)-3-amino-1-{[(2S)-1-(ethylamino)-3-methyl-1-oxo-2-butanyl]amino}-1-oxo-2-propanyl]amino}-4-methyl-2-pentanyl]-L-norvalinamide

Note: "—" represented "not detected", "N" represented "N-potato onion", "S" represented "S-potato onion"

Table S2 The peak heights of all biological replicates of 15 different compounds

Compounds	Peak heights (Response value)															
	N								S							
	1	2	3	4	5	6	7	Average	1	2	3	4	5	6	7	Average
2,4,6-Pyridinetricarboxylic acid	—	—	—	—	—	—	—	—	261651.68	346460.05	311773.29	330080.72	292983.86	309569.07	353178.65	315099.62
4-(trans-4-Pentylcyclohexyl)phenol	521688.08	510976.86	592257.11	492298.93	536072.06	520920.14	491312.30	523646.50	—	—	—	—	—	—	—	—
Cevane-3,6-diol								—	93844.02	105364.15	93265.98	99324.52	97860.33	99174.46	82149.46	95854.70
4-(4-Octylphenyl)-4-oxobutanoic acid	456153.79	452715.47	473658.98	452196.27	470170.99	446763.24	442140.32	456257.00	—	—	—	—	—	—	—	—
2-Amino-2-oxoethyl adamantan-1-ylacetate	—	—	—	—	—	—	—	—	204127.30	217874.29	185818.02	192501.8	189916.94	196948.56	284851.5	210291.20
1-(4-Ethyl-1-piperazinyl)-1-dodecanone	—	—	—	—	—	—	—	—	110695.51	118988.78	104535.28	113510.99	120750.62	116370.64	101529.45	112340.18
6,7,12,13-Tetrahydro-5H-indeno[2,1-a]pyrrolo[3,4-c]carbazol-5-one	—	—	—	—	—	—	—	—	146870.16	166007.61	149666.77	119396.10	133785.00	118565.47	177716.38	144572.50
N-(2-Methoxyethyl)-1-(3-methyl[1,2,4]triazolo[4,3-b]pyridazin-6-yl)-4-	61127.33	50583.95	49315.70	46641.43	51306.11	51214.04	47682.13	51124.38	—	—	—	—	—	—	—	—

piperidinecarboxamide																
(3aS,5S,6aS)-5,6a-Diethyl-5-[(5E)-4-ethyl-5-octen-1-yl]tetrahydrofuro[3,2-b]furan-2(3H)-one	—	—	—	—	—	—	—	—	408101.18	507497.53	440699.48	421646.62	358010.13	378834.26	899306.56	487727.97
{2-[(4aR,6R)-2,3,4,4a,6,6a,7,8,9,10b-Decahydro-1H-benzo[c]chromen-6-yl]phenoxy}acetic acid ammoniate	258239.05	290122.07	287778.88	260481.34	268086.44	275505.46	293320.62	276219.12	—	—	—	—	—	—	—	—
1-(1-Azepanyl)-3-{1-[(4-methyl-1-piperazinyl)methyl]-1H-indol-3-yl}-1-propanone	218001.64	214172.90	225369.28	243845.91	271302.20	267426.37	289765.01	247126.19	—	—	—	—	—	—	—	—
Bis(4-butylphenyl) isophthalate	105751.25	56326.254	63226.135	112017.88	114298.38	102943.15	139831.16	99199.17	—	—	—	—	—	—	—	—
Benzophenone, 4-hexadecyloxy-2-hydroxy	957256.35	718235.66	611080.19	937314.05	1005674.43	847673.38	1291984.91	909888.42	—	—	—	—	—	—	—	—
Methyl 6-O-[2,3,4-tris-O-(2,2-dimethylpropanoyl)-6-methyl-β-D-glucopyranuronosyl]-β-D-galactopyranoside	339956.44	313584.29	259986.08	301114.81	245929.49	229289.12	219445.46	272757.96	—	—	—	—	—	—	—	—
N-(1-Oxidoisonicotinoyl)-L-isoleucyl-N-[(2S)-1-{[(2S)-3-amino-1-{[(2S)-1-(ethylamino)-3-methyl-1-oxo-2-butanyl]amino}-1-oxo-2-propanyl]amino}-4-methyl-2-pentanyl]-L-norvalinamide	1142688.47	1174659.45	1076466.39	964955.63	966248.19	917266.67	1017592.99	1037125.40	—	—	—	—	—	—	—	—
